# Comparative Phylogenetic Analysis for *Aerides* (Aeridinae, Orchidaceae) Based on Six Complete Plastid Genomes

**DOI:** 10.3390/ijms241512473

**Published:** 2023-08-05

**Authors:** Jinliao Chen, Fei Wang, Chengyuan Zhou, Sagheer Ahmad, Yuzhen Zhou, Minghe Li, Zhongjian Liu, Donghui Peng

**Affiliations:** Key Laboratory of National Forestry and Grassland Administration for Orchid Conservation and Utilization at Landscape Architecture and Arts, Fujian Agriculture and Forestry University, Fuzhou 350002, China

**Keywords:** *Aerides*, Aeridinae, plastid genome, phylogenetic analysis

## Abstract

*Aerides* Lour. (Orchidaceae, Aeridinae) is a group of epiphytic orchids with high ornamental value, mainly distributed in tropical and subtropical forests, that comprises approximately 20 species. The species are of great value in floriculture and garden designing because of their beautiful flower shapes and colors. Although the morphological boundaries of *Aerides* are clearly defined, the relationship between *Aerides* and other closely related genera is still ambiguous in terms of phylogeny. To better understand their phylogenetic relationships, this study used next-generation sequencing technology to investigate the phylogeny and DNA barcoding of this taxonomic unit using genetic information from six *Aerides* plastid genomes. The quadripartite-structure plastomes ranged from 147,244 bp to 148,391 bp and included 120 genes. Among them, 74 were protein coding genes, 38 were tRNA genes and 8 were rRNA genes, while the *ndh* genes were pseudogenized or lost. Four non-coding mutational hotspots (*rpl20–rpl33, psbM, petB, rpoB–trnC^GCA^*, Pi > 0.06) were identified. A total of 71–77 SSRs and 19–46 long repeats (>30 bp) were recognized in *Aerides* plastomes, which were mostly located in the large single-copy region. Phylogenetic analysis indicated that *Aerides* was monophylic and sister to *Renanthera*. Moreover, our results confirmed that six *Aerides* species can be divided into three major clades. These findings provide assistance for species identification and DNA barcoding investigation in *Aerides,* as well as contributes to future research on the phylogenomics of Orchidaceae.

## 1. Introduction

*Aerides* Lour. (Aeridinae, Vandeae and Orchidaceae) consists of approximately 20 species of epiphytic and lithophytic perennial herbs [1], which are mainly distributed in the tropical and subtropical regions of Asia [2]. The members of *Aerides* produce a spectacular branched inflorescence containing numerous pinkish and scented flowers [3]. These orchids are used by the orchid breeders to produce numerous artificial hybrids and cultivars. Since the establishment of *Aerides* by Loureiro in 1790, the genus has undergone multiple intra-generic taxonomic revisions [2,3,4,5]. Lindley (1840) [4] and Pfitzer (1887) [5] introduced five sections of *Aerides*, including 26 and 15 species, respectively. Christenson (1987) used morphological analysis to subdivide the genus into four clades (*Aerides*, *Falcata*, *Fieldingia*, *Rubescens*) with a total of 19 species and described the subdivision characteristics of each clade in detail [6]. Although the morphological boundaries of the *Aerides* have been clarified, the relationship between *Aerides* and other closely related genera is still ambiguous in terms of phylogeny.

The constant enrichment of molecular evidence for phylogenetic relationships is an ongoing outcome of the advancements in molecular biology. Kocyan (2008) used three markers such as *ITS, matK* and *trnL-F* to study the molecular phylogeny of *Aerides* and showed that the genus was monophyletic and subdivided it into three evolutionary clades (i.e., sect. *Fieldingia*, sect. *Aerides* and sect. *Crispa*) [3]. However, Christenson (1987) found that only some clades were basically consistent. Comparing these clades with the four clades established based on morphology [6], Xiang (2012) found that the *Aerides* had multiple origins: *A. odorata* and *A. flabellate* and *Ascocentrum ampullaceum* and *Vanda* are sister groups [7]. In addition, *A. thibautiana* and *A. krabiensis* were separated into a monophyletic group. In order to better understand their phylogeny, it is necessary to determine the differences in genetic information among the major lineages of the *Aerides*.

With the development of next-generation sequencing (NGS) technology, plastid genome data contributed to the deeper comprehension of the phylogenetic relationships among numerous complex orchid taxa [8,9]. Tu (2020) used the plastid genomes of 46 Goodyerinae plants to reveal the molecular location of the *Cheirostylis* and *Goodyera* clades within the Goodyerinae [10]. Liu (2020) used plastid genome sequencing to explain phylogenetic relationships among members of the *Cleisostoma–Gastrochilus* clades in Aeridinae but did not include *Aerides* members [11].

To comprehensively understand the evolution of the plastid genomes of *Aerides*, six species were collected in this study. The plastid structure, sequence differences, mutation hotspots and repeat regions were characterized, variable sites were identified, and the phylogenetic relationships were revealed to further elucidate the evolutionary pattern at the plastid genome level of *Aerides*. Therefore, our study provides valuable information on the phylogenetic relationships and species identification of *Aerides*.

## 2. Results

### 2.1. Characteristics of the Plastome

**Figure 1:**
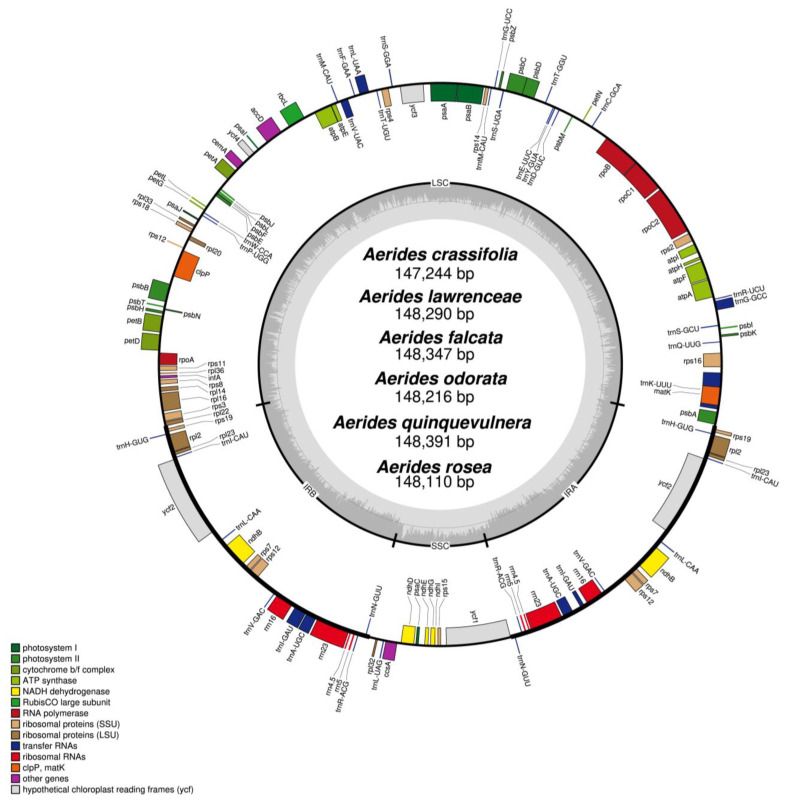
The annotation map of six *Aerides* plastomes. The darker gray in the inner circle corresponds to the GC content. The IRa and IRb (two inverted repeating regions), LSC (large single-copy region) and SSC (small single-copy region) are indicated outside of the GC content.

The genome sizes of the six newly sequenced plastomes of *Aerides* ranged from 147,244 bp (*A. crassifolia*) to 148,391 bp (*A. quinquevulnera*) (Figure 1), which were in the range of common angiosperm plastome sizes. The quadripartite structure with two inverted repeat regions (IRs) exhibited a large single-copy region (LSC) and a small single-copy region (SSC). The size of each region varied across all species (Table 1). For the IR region, the sizes ranged from 25,706 bp (*A. odorata*) to 25,852 bp (*A. rosea*). The SSC regions were 11,033 bp (*A. rosea*) to 11,897 bp (*A. crassifolia*), and the LSC regions were in the range of 83,913 bp (*A. crassifolia*) to 85,570 bp (*A. falcata*). The G/C content was approximately 36.8% (Table 1).

The plastomes of *Aerides* encoded 120 genes (including repetitive genes), including 74 protein-coding genes, 38 transfer RNA (tRNA) genes and eight ribosomal RNA (rRNA) genes (Table 1). The *ndh* genes were all pseudogenes with 5–8 members in each plastome (Table 1). The plastomes of *A. falcata* and *A. odorata* contained eight (*ndhB/C/D/E/G/I/J/K*) pseudogenes; *A. lawrenceae* and *A. quinquevulnera* possessed seven (*ndhB/C/D/E/G/J/K*) pseudogenes; *A. rosea* and *A. crassifolia* possessed six (*ndhB/C/D/G/J/K*) and five (*ndhB/D/E/G/I*) pseudogenes, respectively.

**Figure 2:**
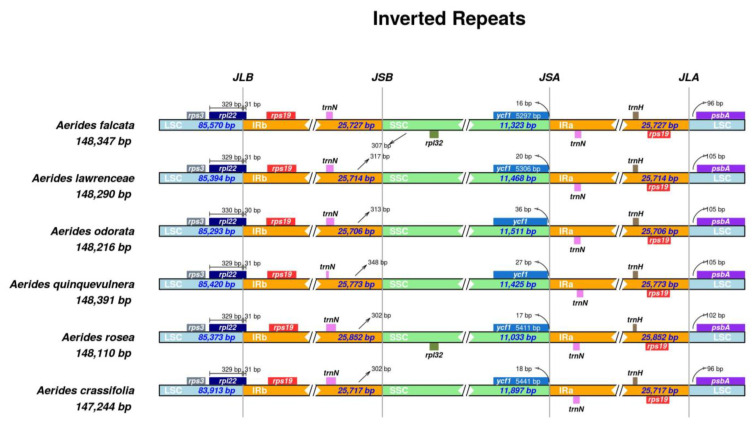
Comparison of junctions between the LSC, SSC and IR regions among six newly assembled *Aerides* plastomes.

The positions of the IR junctions were well-conserved across the six species of *Aerides* (Figure 2). At the junction between the LSC and IRb (JLB), the *rpl22* genes of the LSC crossed over into IRb. The adjacent regions of LSC and IRa (JLA), which were located in the *rps19* genes and *psbA* genes, were similar in *Aerides*. The *rpl32* and *trnN* genes were adjacent to the junction between SSC and IRb (JSB), while the *trnN* and *ycf1* genes were adjacent to the junction between SSC and IRa (JSA). The *ycf1* genes were complete in the SSC region.

### 2.2. Repeated Analysis

**Figure 3:**
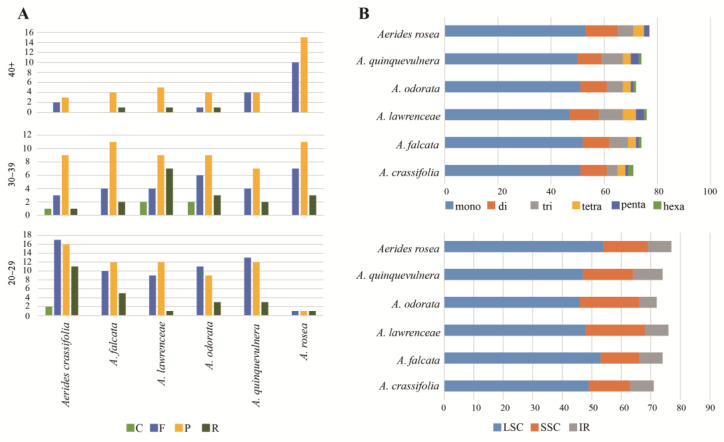
Summary of the simple sequence repeats (SSR) across the *Aerides* species. (**A**) Variation in repeat abundance and type in six plastomes. (**B**) Number of SSRs for each *Aerides* species by SSR unit size and number of SSRs for each *Aerides* species by location in IR, LSC and SSC.

The number and distribution area of SSRs were analyzed to elucidate allied species or intra-species variations. The long repeats of *Aerides* plastomes, including the complement (C), forward (F), palindrome (P) and reverse (R) types, were analyzed by the online REPuter program [12] (Figure 3A, Supplementary Table S2). The number of large repeats detected in the six plastomes were 49 (*A. falcata, A. odorata, A. quinquevulnera* and *A. rosea*), 50 (*A. lawrenceae*) and 65 (*A. crassifolia*), respectively. Except for *A. rosea*, almost all the repeats ranged from 20 to 39 bp, with the fewest in 40–49 bp. No complement repeats were detected above 40 bp in length, and they were rare even in the smaller size ranges. In the 30–39 bp group, complement and reverse repeats were found in a few species (Figure 3A).

We also analyzed regions in mononucleotide, dinucleotide, trinucleotide, tetranucleotide, pentanucleotide, and hexanucleotide SSR. *Aerides* had a total of 71 (*A. crassifolia*)–77 (*A. rosea*) SSRs (Figure 3B, Supplementary Table S3). Among the SSRs, the mononucleotide repeats were the most abundant. At least 47–53 mononucleotide repeats were found in the six species, followed by the dinucleotides (9–12 repeats), trinucleotides (4–9 repeats), tetranucleotides (3–5 repeats) and pentanucleotide repeats (1–3 repeats). For hexanucleotide repeats, all the species were recorded with 1–2 repeats, except *A. rosea*, which had no repeats. Most SSRs were located in the LSC region, whereas few SSRs were located in the IR region (Figure 3B, Supplementary Table S3).

### 2.3. Plastome Sequence Divergence and Barcoding Investigation

**Figure 4:**
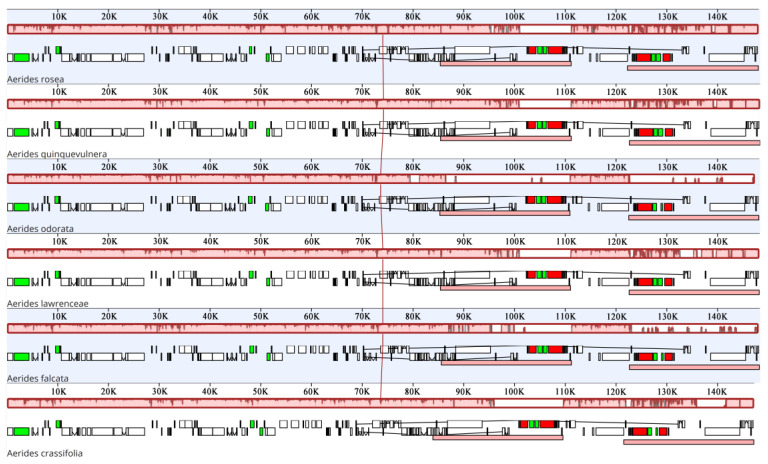
Plastome comparison of six species of *Aerides* using a progressive MAUVE algorithm.

**Figure 5:**
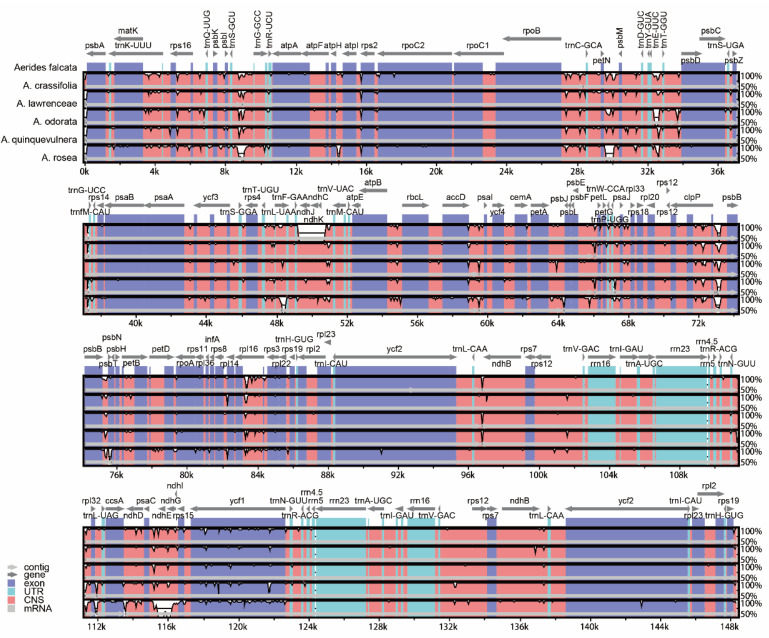
Global alignment of six *Aerides* plastomes using mVISTA with *A. falcata* as reference. The *y*-axis shows the coordinates between the plastomes.

A comparative analysis of the complete plastomes can reveal differences between different species (Figure 4 and Figure 5). We found that the plastome sequences of *Aerides* exhibited a high degree of similarity, and no rearrangement occurred. The intergenic and intragenic regions were found to have the least similarity between plastomes in *Aerides* (Figure 5), especially in LSC regions (from *psb1* to *trnG^GCU^*, *rpoB* to *psbD* and *trnF^GAA^-trnV^UAC^*) and SSC regions (from *rpl32* to *ycf1*). Given these results, there are many intergenic and intragenic regions to develop DNA barcodes to differentiate *Aerides* species.

**Figure 6:**
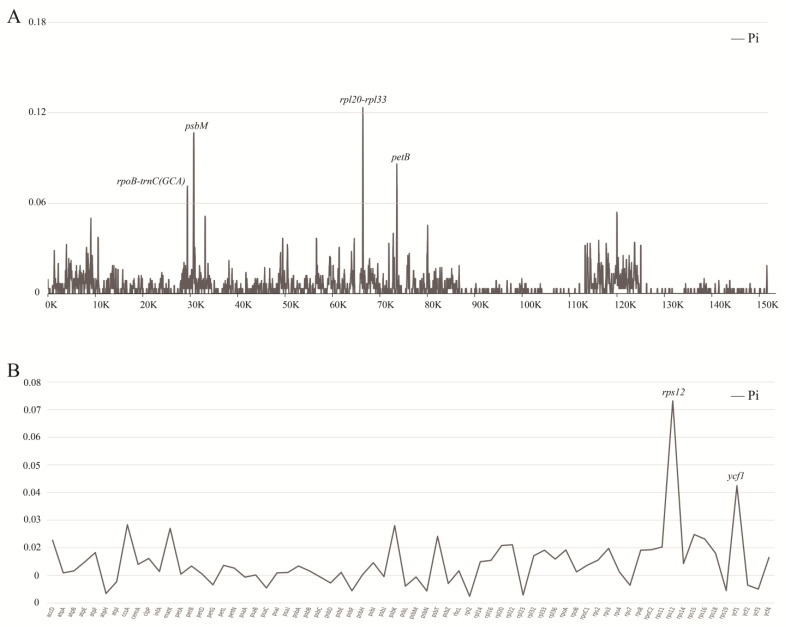
Sliding window test of nucleotide diversity (Pi) in the *Aerides* plastomes. (**A**) The nucleotide diversity of the complete plastome and four mutation hotspot regions (Pi > 0.06) were annotated. (**B**) The nucleotide diversity of 68 protein coding sequences showing two mutation hotspot regions (Pi > 0.03). The window size was set to 100 bp and the sliding window size was 25 bp. *x* -axis, position of the midpoint of a window; *y* -axis, Pi values of each window.

To further analyze the mutational hotspots of *Aerides* plastomes, we used DnaSP6 to analyze the nucleotide diversity (Pi) for the alignment of complete genome (Figure 6, Supplementary Tables S4 and S5). The nucleotide diversity (Pi) values of the six plastomes ranged from 0 to 0.12333. At the cutoff point of Pi > 0.06, we selected four mutational hotspots (*rpl20-rpl33 > psbM > petB > rpoB-trnC^GCA^*) as candidate barcodes. Protein-coding genes were also used in nucleotide diversity analysis. The results showed that at the cutoff point of Pi > 0.03, two coding sequences (*rps12 > ycf1*) had high nucleotide diversity and were suitable for phylogeny.

### 2.4. Phylogenetic Analysis

**Figure 7:**
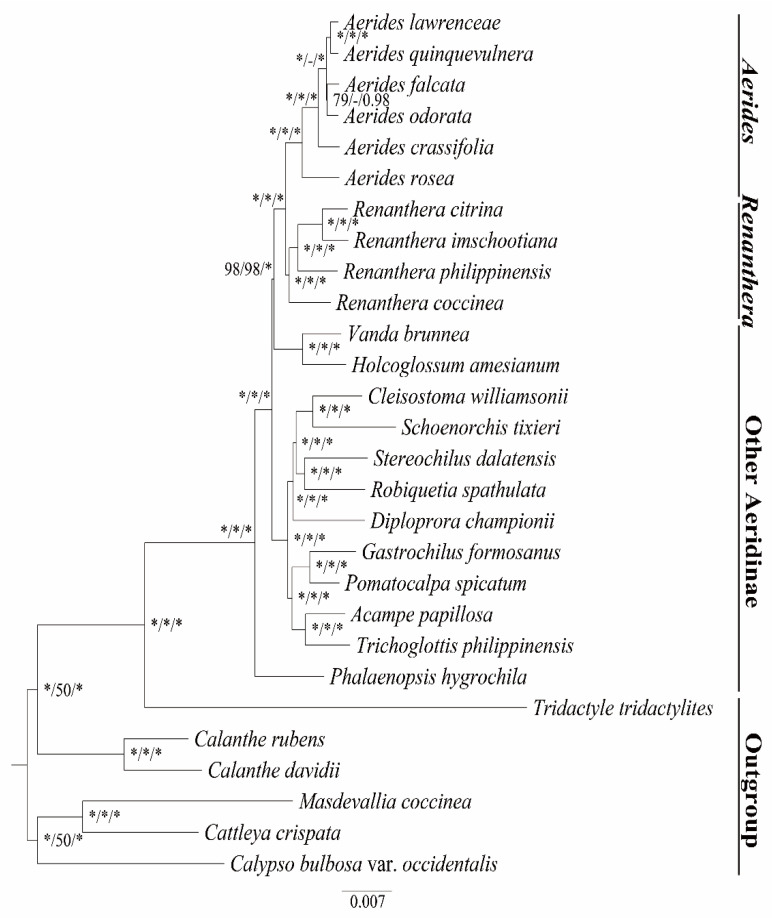
Phylogenetic tree of *Aerides* and the other 16 Aeridinae species based on the complete plastome data. Numbers near the nodes are bootstrap percentages and Bayesian posterior probabilities (BSML left, BSMP middle and PP right). A dash (-) indicates that a node is inconsistent between the topology of the MP/ML trees and the Bayesian tree. An asterisk (*) indicates that the node is 100 bootstrap percentage or 1.00 posterior probability.

The phylogenetic relationships inferred by ML analysis (IQ-Tree Ultrafast Method) of the complete genomes and 68 protein-coding genes predicted the same topology (Figure 7; Supplementary Figure S1). In general, the phylogenetic relationship within *Renanthera* was well-resolved (BS ≥ 75%, PP ≥ 0.90). Six species of *Aerides* formed a monophyletic genus, and they were classified into three major clades. *A. rosea* formed the first clade of *Aerides*, and *A. crassifolia* formed the second clade. *A. lawrenceae*, *A. quinquevulnera*, *A. falcata* and *A. odorata* formed the third clade. All the branch nodes in the phylogenetic tree were strongly supported by the ML analysis and the BI analysis.

## 3. Discussion

### 3.1. The Plastome Characteristics and Structural Evolution

The size of the plastid genome of Orchidaceae has a large variation depending on the different life types, ranging from 19,047 bp (*Epipogium roseum*) [13] to 212,688 bp (*Cypripedium subtropicum*) [14]. The plastid genome size of *E. roseum* is mainly the result of the loss in genes associated with the fungal heterotrophic habitat, while the plastid genome size of *C. subtropicum* is due to the expansion of non-coding regions [13,14,15]. Currently, the published plastids of Aeridinae members range from 142,859 bp (*Schoenorchis seidenfadenii*) [11] to 149,689 bp (*Thrixspermum tsii*) [16]. In this study, we obtained the plastome sequences of six species of *Aerides* using next-generation sequencing technology. The plastome size ranges from 147,244 bp to 148,391 bp, wherein the structure and gene order are highly conserved. Our result is in line with the sizes of the previously reported orchid plastomes [11,13,14,15,16,17].

In the majority of angiosperms, plastid genomes are typically inherited maternally and exhibit minimal recombination, thereby maintaining a highly conserved structure among closely related species [18]. The previous studies have shown that contraction and expansion of the IR region is a common phenomenon in the evolution process [19], which is the main reason for differences in plastome genome length [20,21]. Such variations can occur at the boundaries of inverted repeats (IRs) and single-copy regions (LSC and SSC), allowing certain genes into IR or SC regions. It is also the main reason for the difference in plastome length [20]. In this study, we observed slight difference in the IR/SC boundary regions of *Aerides* plastomes. For example, the 3′ end of the *rpl22* gene of all six species of *Aerides*, which should be intact in the LSC region, extended into the IRb region for 30–31 bp, which was found in *Renanthera* [22]. In addition, the *ycf1* gene in the SSC region of other orchids (such as *Pholidota* [23] and *Thuniopsis* [24]) was observed crossing over JSA, extending into the IRa region. However, the *ycf1* of *Aerides* was intact in the SSC region. Our study indicates that the plastid genome size and the IR regions are conserved in *Aerides* compared with other orchids. Therefore, the difference in plastid genome size in *Aerides* may be due to the presence of indels in the intergenic spacer regions and the loss of pseudogenization in the *ndh* genes [14,17]. However, the total GC content of *Aerides* does not exceed 36.8%, which is similar to other members of Aeridinae.

There are 11 *ndh* genes in the plastids of plants [25], and we detected 5-8 *ndh* genes in six *Areides* plants, and all of them were pseudogenized, while the *ndh A/F/H* genes were completely lost. This is consistent with the plastome study of the subtribe Aeridinae [11,26]. Although the *ndh* genes were detected in the mitochondrial (mt) genome of some orchids, there is no direct evidence that these genes are associated with the loss of *ndh* genes in the plastome [27]. Therefore, the mechanism of *ndh* gene deletion and pseudogenization in orchids needs to be explored in further studies. In addition, studies have suggested that the loss of *ndh* genes in angiosperms can prevent plants from evolving and diversifying [28,29] and reduce the ecological adaptability of species. The deletion of *ndh* genes was found in all five subfamilies of Orchidaceae, with epiphytic orchids showing a higher frequency of *ndh* gene loss than geophytic orchids [15]. However, this does not directly prove that the diversity and adaptability of Orchidaceae are related to the loss of *ndh* genes.

### 3.2. The Barcoding Investigation and Phylogenetic Analysis

Nucleotide diversity (Pi) can indicate the degree of variation in the nucleic acid sequences in different species, and the position with higher variability can be used as a molecular marker of population genetics [30,31]. The genetic loci used for DNA barcoding usually contain enough informative loci to effectively define closely related species, which was demonstrated in orchids [32,33,34,35]. In this study, a nucleotide diversity analysis was performed on intact plastids of *Aerides,* and four highly variable regions were identified. The nucleotide diversity analysis of protein-coding genes identified two highly variable coding genes. The six highly variable regions identified in this study can be used as DNA molecular markers to distinguish *Aerides* relatives, and the results can be used to develop Aeridinae DNA barcodes. Repeated sequences play an important role in the evolution of species, as well as the inheritance and variation of genes within species [36,37]. These repetitive sequences are widely used in the studies on genetic diversity, population structure, and closely related species identification [38,39,40,41]. In this study, a total of 71–77 SSRs and 19–46 long repeats (>30 bp) were identified from *Aerides* plasmids, indicating that the plasmid genome of *Aerides* retained abundant genetic information. Most of the SSRs are mononucleotide repeats in these six *Aerides* species, which are mostly located in the intergeneric regions of LSC and enriched in non-coding regions. Similar results are found in most angiosperms [42,43,44,45]. The above findings can provide a data basis for further studies on population genetics.

Our results revealed the phylogenetic position of *Aerides* and the intrageneric relationships. Despite being a valuable and threatened class of orchids, there are few studies on the phylogenetic relationships in *Aerides*. According to phylogenetic analysis using short gene sequences, *Aerides* is monophyletic, sisterly to *Renanthera*, *Arachnis* and *Esmeralda* [46]. However, their phylogenetic relationships predicted unstable topology and low support values based on the small amount of short gene sequence data. The role of plastome data in the phylogenetic relationships in the reconstruction of tribes, subtribes and genera in Orchidaceae was also demonstrated [10,11,32]. Our results suggest that the *Aerides* clade is an independent clade of Aeridinae that is sister to the *Renanthera* clade. Similar results were found in previous studies on short gene sequences [46]. Nevertheless, the *Aerides-Renanthera* clade is sister to the *Vanda* clade, which is inconsistent compared with the previous studies [46]. This study highly signifies the intrageneric consanguinity of *Aerides* and provides a new perspective for elucidating their relationships.

## 4. Materials and Methods

### 4.1. Plant Materials, DNA Extraction and Sequencing

Six *Aerides* species were selected, including *A. falcata*, *A. crassifolia*, *A. odorata*, *A. quinquevulnera*, *A. rosea* and *A. lawrenceae*. They were introduced and cultivated in the Fujian Agriculture and Forestry University, Fujian province, China. Their voucher information is given in Supplementary Table S1. The total DNA was isolated using a modified CTAB method [47]. Short-insert (500 bp) pair-end (PE) libraries were constructed, and the sequencing was conducted by the Beijing Genomics Institute (Shenzhen, China) on the Illumina HiSeq 2500 platform with a read length of 150 bp. At least 10 Gb of clean data were obtained for each species.

### 4.2. Plastome Assembly and Annotation

The plastome assembly and annotation were performed following the previously described methods [9]. In short, The paired-end reads were assembled using the GetOrganelle pipeline (https://github.com/Kinggerm/GetOrganelle, accessed on 30 April 2023), and then, the filtered reads were assembled by SPAdes version 3.10 [48]. The published plastome of *Phalaenopsis hygrochila* (MN124430) was used as a reference for the assembly of plastomes. The gene annotation was carried out using DOGMA [49] based on default parameters and checked through Geneious Prime v2021.1.1 [50]. The circle maps were drawn using OGDRAW [51].

### 4.3. Genome Comparison and Analysis, IR Border and Divergence Analyses

The plastome genomes across the six species of *Aerides* were aligned with mVISTA using the LAGAN alignment program [52] with the sequence of *A. falcata* as a reference. The rearrangements of plastomes were detected and plotted using Mauve of six species [53]. The boundaries between the IRs, SSC and LSC of the plastomes were compared using the online program IRscope (https://irscope.shinyapps.io/irapp/, accessed on 30 April 2023) [54].

To identify the mutational hotspot regions and genes, the plastome sequences were aligned using MAFFT v7 [55]. Then, the nucleotide diversity (Pi) of six plastomes of *Aerides* was calculated using DnaSP v6.12.03 (DNA Sequences Polymorphism) [56]. Highly mutational hotspot regions were identified through a sliding window strategy. The step size was set to 25 bp, with a 100 bp window length.

### 4.4. Repeat Sequence Analysis

The online software REPuter (https://bibiserv.cebitec.uni-bielefeld.de/reputer, accessed on 30 April 2023) was used to identify the repeat sequences, including forward, palindrome, reverse, and complement long repeats. The maximum and minimum repeat sizes were set as 50 bp and 20 bp, respectively; while the hamming distance was set to 3 [12]. MISA-web was used to detect simple sequence repeats (SSRs). The parameters were set as a threshold of mononucleotide, dinucleotide, trinucleotide, tetranucleotide, pentanucleotide and hexanucleotide SSRs and minimal repeat numbers of 10, 5, 4,3, 3 and 3, respectively [57].

### 4.5. Phylogenetic Reconstruction

We used the whole plastome and 68 protein-coding sequences to perform the phylogenetic analysis of 28 species of Orchidaceae. Of these 28 species, 6 Aerides species are our newly sequenced species and the other 22 species of 18 genera are from the complete plastid data publicly available at NCBI. A list of the taxa analyzed with voucher information and GenBank accessions is provided in Supplementary Table S1. The whole plastome sequences were aligned by Geneious Prime v2021.1.1 [50]. A total of 68 protein-coding genes were aligned by PhyloSuite v1.2.2 [58]. The phylogenetic relationships were analyzed by maximum parsimony (MP), maximum likelihood (ML) and Bayesian inference (BI) on the website CIPRES Science Gateway [59]. All characters were equally weighted and unordered, and a heuristic search with 1000 random addition sequence replicates and TBR branch swapping was performed. For ML analysis, the GTRCAT model was specified for all datasets and 1000 repeated self-expanding analyses were performed [60].

The Bayesian analyses were performed with MrBayes v. 3.2.6 [61], and four Markov chains were run for 10,000,000 generations, with one tree sampled every 100 generations. The first 25% of trees were discarded as burn-in samples to ensure that each chain reaches a stable state and the posterior probabilities (PP) were estimated.

## 5. Conclusions

Our research shows that the overall structure and gene content of the plastomes of six *Aerides* species are relatively conserved, with only certain differences in genome size, gene content, GC content, repeat sequences and IR boundary, and all *ndh* genes were lost or pseudogenized. This study provides a reference for developing DNA barcoding in further studies on the *Aerides* species. The phylogenetic analyses based on the available data identified the genus *Aerides* as a separate clade of Aeridinae, sister to *Renanthera*, thereby significantly aiding in reconstructing the phylogenetic connections of Aeridinae. Therefore, our findings offer valuable support for future investigations into the phylogeny and evolution of *Aerides* and Orchidaceae.

## Figures and Tables

**Table 1:** Characteristics of the complete plastomes of the *Aerides* lineages.

Species Name	Size (bp)	GC Content (%)	LSC Size in bp (%)	IR Size in bp (%)	SSC Size in bp (%)	Total Number of Gene	Protein-Encoding Gene	tRNA Gene	rRNA Gene	Number of *ndh* Fragment
*Aerides* *crassifolia*	147,244	36.8	83,913 (57.0)	25,717 (34.9)	11,897 (8.1)	120	74	38	8	6
*A. falcata*	148,347	36.8	85,570 (57.7)	25,727 (34.7)	11,323 (7.6)	120	74	38	8	9
*A. lawrenceae*	148,290	36.8	85,394 (57.6)	25,714 (34.7)	11,468 (7.7)	120	74	38	8	8
*A. odorata*	148,216	36.8	85,293 (57.5)	25,706 (34.7)	11,511 (7.8)	120	74	38	8	9
*A. quinquevulnera*	148,391	36.7	85,420 (57.6)	25,773 (34.7)	11,425 (7.7)	120	74	38	8	8
*A. rosea*	148,110	36.7	85,373 (57.6)	25,852 (34.9)	11,033 (7.5)	120	74	38	8	7

## Data Availability

The six plastome sequences are deposited in GenBank of the NCBI repository, accession numbers OR159896 to OR159901.

## Supplementary Materials

The following supporting information can be downloaded at: https://www.mdpi.com/article/10.3390/ijms241512473/s1.
