# Supplementary material for: Comparative Phylogenetic Analysis for Aerides (Aeridinae, Orchidaceae) Based on Six Complete Plastid Genomes
Source: Int J Mol Sci. 2023 Aug 5;24(15):12473. doi: 10.3390/ijms241512473 (PMC10420012; doi:10.3390/ijms241512473)
Supplement: Supplementary file 1 [file ijms-24-12473-s001.zip › ijms-2460091-supplementary/Aerides Supplementary Figures and Tables-with revised marker/Supplementary Figures and Tables.pdf]

Supplementary Figure 1. Phylogenetic analysis of 22 Aeridinae species based on 68 protein coding genes.

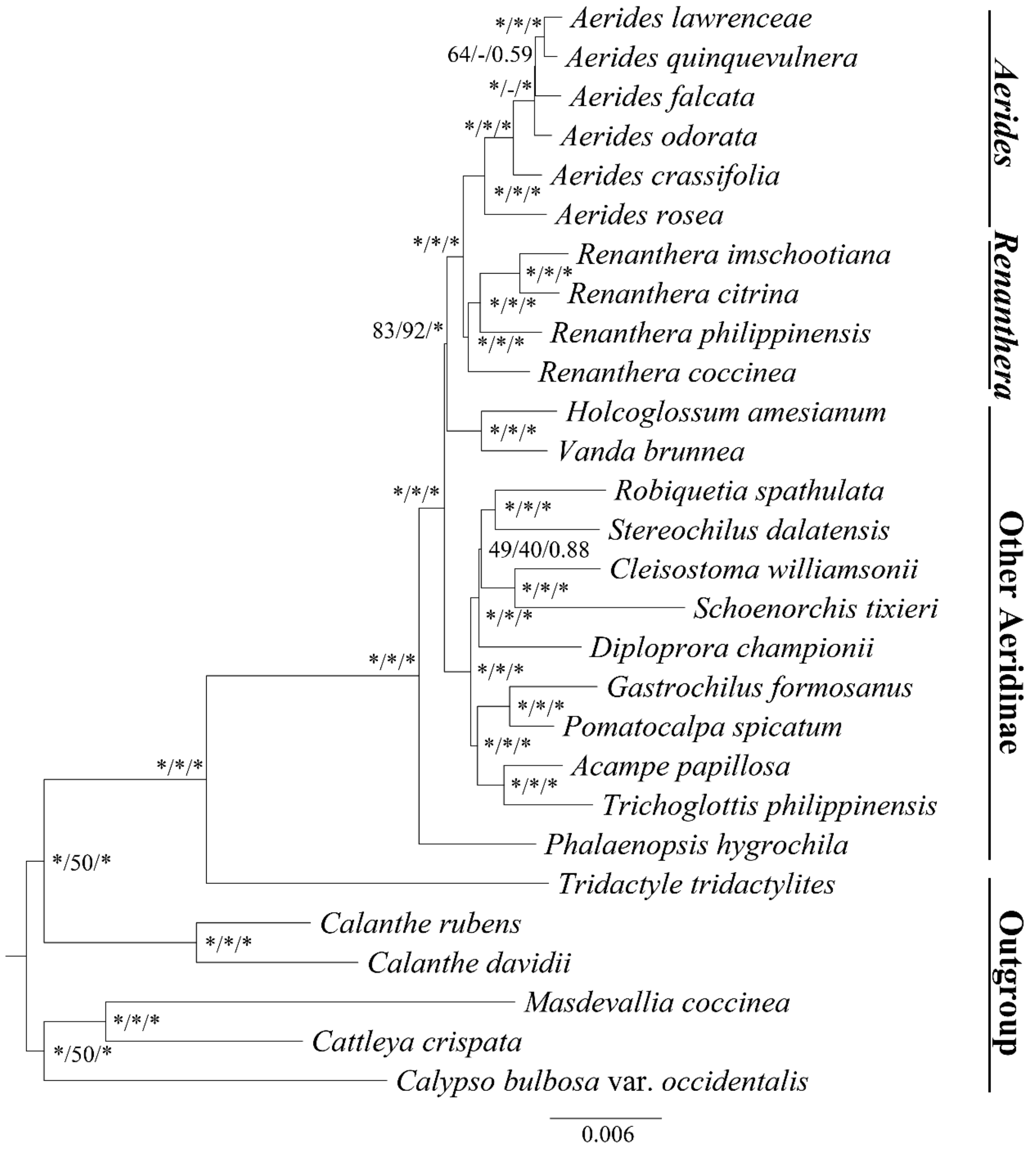

Supplementary Figure 1. Phylogenetic analysis of 22 Aeridinae species based on 68 protein coding genes. Numbers near the nodes are bootstrap percentages and Bayesian posterior probabilities (BSML left, BSMP middle, and PP right). - indicates that a node is inconsistent between the topology of the MP/ML trees and the Bayesian tree. * indicates that the node is 100 bootstrap percentage or 1.00 posterior probability.

Supplementary Table 1 Source and voucher information for this study. Voucher specimens were deposited in the herbariums of Forestry College of Fujian Agriculture and Forestry University (FJFC) and National Center for Biotechnology Information (NCBI).

Species	Source	Voucher	GenBank accession numbers
Aerides lawrenceae.	This study		OR159898
Aerides odorata	This study		OR159899
Aerides quinquevulnera.	This study		OR159900
Aerides crassifolia	This study		OR159897
Aerides falcata	This study		OR159896
Aerides rosea.	This study		OR159901
Acampe papillosa	Liu et al., 2020	Liu3246	MN124418
Cleisostoma williamsonii	Liu et al., 2020	Liu4525	MN124426
Diploprora championii	Liu et al., 2020	Liu4480	MN124409
Gastrochilus formosanus	Liu et al., 2020	Liu4265	MN124435
Holcoglossum amesianum	Li et al., 2019	9419	NC041511
Phalaenopsis hygrochila	Liu et al., 2020	Liu3336	MN124430
Pomatocalpa spicatum	Liu et al., 2020	Liu4589	MN124411
Renanthera citrina	Zhang et al., 2021	OR-001	OK377033
Renanthera coccinea	Zhang et al., 2021	OR-002	OK377034
Renanthera imschootiana	Zhang et al., 2021	OR-003	OK377035
Renanthera philippinensis	Zhang et al., 2021	OR-004	OK377036
Robiquetia spathulata	Liu et al., 2020	Liu5248	MN124410
Schoenorchis tixieri	Liu et al., 2020	Liu4123	MN124407
Stereochilus dalatensis	Liu et al., 2020	Lior003	MN124431
Trichoglottis philippinensis	Liu et al., 2020	Lior075	MN124404
Vanda brunnea	Li et al., 2019	13059	NC041522
**Outgroup**			
Calanthe davidii	Dong et al., 2018	-	MG925365
Calanthe triplicata	Yang et al., 2014	-	KF753635
Calypso bulbosa var. *occidentalis*	Barrett et al., 2018	CFB 349 OR	MG874037
Cattleya crispata	da Rocha et al., 2016	-	KP168671
Masdevallia coccinea	Kim et al., 2015	-	KP205432
Tridactyle tridactylites	D'haijère et al., 2022	-	MW760855

Supplementary Table 2. The details information of long repeats.

seq len	species	*A. crassifolia*	*A. falcata*	*A. lawrenceae*	*A. odorata*	*A. quinquevulnera*	*A. rosea*
40+	Complement	0	0	0	0	0	0
	Forward	2	0	0	1	4	10
	Palindrome	3	4	5	4	4	15
	Reverse	0	1	1	1	0	0
30-39	Forward	3	4	4	6	4	7
	Palindrome	9	11	9	9	7	11
	Reverse	1	2	7	3	2	3
	Complement	2	0	0	0	0	0
20-29	Forward	17	10	9	11	13	1
	Palindrome	16	12	12	9	12	1
	Reverse	11	5	1	3	3	1

Supplementary Table 3. The details information Small simple repeats.

species	mono	di	tri	tetra	penta	hexa	total
Aerides crassifolia	51	10	4	3	1	2	71
Aerides falcata	52	10	7	3	1	1	74
Aerides lawrenceae	47	11	9	5	3	1	76
Aerides odorata	51	10	6	3	1	1	72
Aerides quinquevulnera	50	9	8	3	3	1	74
Aerides rosea	53	12	6	4	2	0	77

species	LSC	SSC	IR	total
Aerides crassifolia	49	14	8	71
Aerides falcata	53	13	8	74
Aerides lawrenceae	48	20	8	76
Aerides odorata	46	20	6	72
Aerides quinquevulnera	47	17	10	74
Aerides rosea	54	15	8	77

Supplementary Table 4. The nucleotide diversity of six *Aerides* plastome.

Supplementary Table 5. The nucleotide diversity of 68 protein coding genes in *Aerides*.
