# Supplementary figures and images for: Comparative Phylogenetic Analysis for Aerides (Aeridinae, Orchidaceae) Based on Six Complete Plastid Genomes
Source: Int J Mol Sci. 2023 Aug 5;24(15):12473. doi: 10.3390/ijms241512473 (PMC10420012; doi:10.3390/ijms241512473)

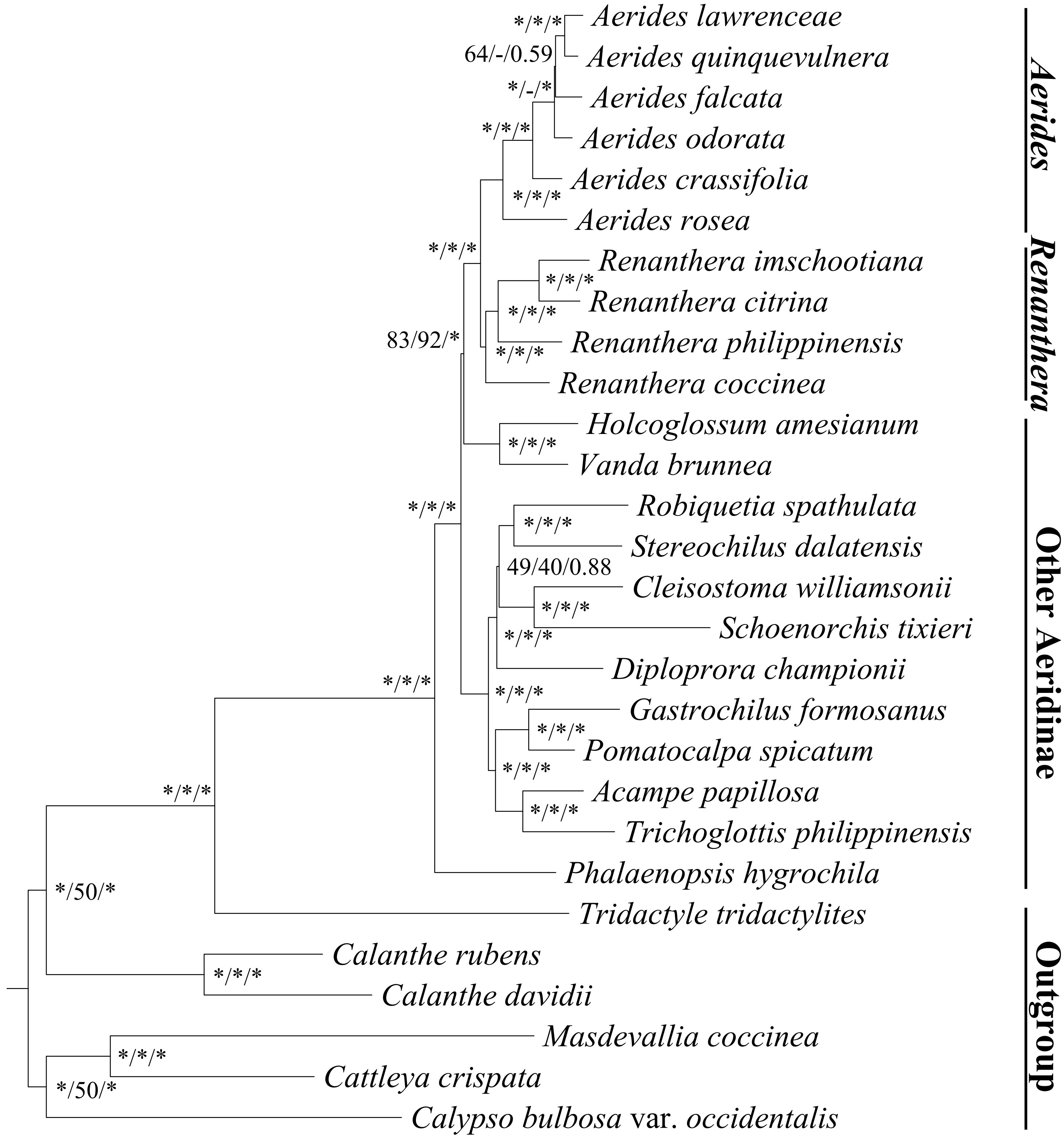

Supplement: Supplementary file 1 [file ijms-24-12473-s001.zip › ijms-2460091-supplementary/Aerides Supplementary Figures and Tables-with revised marker/Supplementary Figure 1.pdf]
